# Deciphering the biology of IgG4-related disease: specific antigens and disease?

**DOI:** 10.1136/gutjnl-2017-314861

**Published:** 2017-11-03

**Authors:** Debashis Haldar, Gideon M Hirschfield

**Affiliations:** 1 Centre for Liver Research, NIHR Birmingham Biomedical Research Centre, University of Birmingham, Birmingham, UK; 2 Centre for Rare Diseases, Institute of Translational Medicine, University Hospitals Birmingham NHS Foundation Trust, Birmingham, UK

**Keywords:** antigen presentation, autoimmunity, B cell, immunology, autoimmune disease

Immunoglobulin G4-related disease (IgG4-RD) is the name applied to a corticosteroid and/or B-cell depletion responsive illness, in which patients present with the consequences of usually multiorgan, relapsing and remitting, fibroinflammation.^1^ The disease is histologically characterised by obliterative phlebitis, storiform fibrosis and a dense lymphoplasmacytic infiltrate.^2^ IgG4-RD is not a new disease, but is benefiting from the application of new technologies in the pursuit of better biological understanding. The histologic enrichment of IgG4-expressing plasma cells is a diagnostic hallmark of disease that additionally serves as a biological phenomenon driving scientific evaluation.^3^ Key disease themes have evolved to include a large clonal expansion of activated plasmablasts and CD4+ cytotoxic, inflammatory and profibrotic lymphocytes. Therapeutically, a reduced frequency of CD4+ cytotoxic lymphocytes are seen after B-cell depletion; such therapy may consequently impact on antigen presentation.^4–6^ To date, the activity of IgG4-RD is not readily tracked by disease biomarkers with even serum IgG4 concentration remaining an imperfect diagnostic and prognostic tool for many patients.^7^

IgG4 molecules have structural and functional characteristics suggesting anti-inflammatory and tolerance-inducing effects,^8 9^ and in IgG4-RD a reported oligoclonal reactivity to multiple antigens.^4 10^ In *Gut*, Hubers *et al* describe a body of work that identifies the first IgG4 autoantibody against an antigen which appears to be specific to IgG4-RD (IgG4-associated cholangitis (IAC) and autoimmune pancreatitis (AIP)), at the exclusion of its major differential diagnoses: primary sclerosing cholangitis and cholangiocarcinoma.^11^ The authors demonstrate that patients with IgG4-RD raise IgG1 and IgG4 in their sera that recognise a 56 kDa cytosolic protein in an immortalised cell line lysate (H69 cholangiocytes) and in human liver lysate. Both IgG1 and IgG4 antibodies recognise the same 56 kDa protein, and subsequent label-free quantitative liquid chromatography-tandem mass spectrometry analysis and immunoprecipitation identifies the cytosolic protein, Annexin A11, as the target. Annexin A11 IgG4 antibodies were also found in sera from patients with IgG4-sialadenitis, in the absence of IAC or AIP, suggesting the target antigen to not be site specific.

This work is in the context of next-generation sequencing studies—a technique that has enabled identification of immunoglobulin clones within a restricted repertoire—yielding strong evidence to support an antigen-driven process driving the pathology of IgG4-RD. An examination of circulating plasmablasts in those with active IgG4-RD has found the expanded pool of cells to have undergone class switching to IgG4, to be oligoclonally restricted^4 10 12 13^ and to be subject to extensive somatic hypermutation.^10^ Flow cytometric evaluation of circulating IgG4+ B cells confirms increased numbers of blood IgG4+ memory B cells with reduced expression of CD27 and CXCR5 and increased signs of antibody maturation.^14^ In affected tissue, CD4+ T cells constitute the most abundant cell type, and an analysis of the nature of these cells in disease revealed prominent clonal expansion of CD4+ T cells with a cytolytic phenotype.^5^ These findings in concert strongly suggest an antigen-driven process that requires critical T cell and B cell interaction.^15^

Nevertheless, the number and nature (foreign or self) of the antigens that drive the disease remains a subject for ongoing study, collaboration and cross-validation. Dutch and UK patient questionnaires revealed an association with chronic exposure to industrial dusts, gases, oils, solvents and pesticides in ‘blue-collar’ professionals—though further work to elucidate candidate antigens and causality is important.^16^ A series of prior studies implicated molecular mimicry between antigens on *Helicobacter pylori* and self (eg, α-carbonic anhydrase of *H. pylori* and human carbonic anhydrase II) to drive disease.^17–19^ However, an association between exposure to *H. pylori* infection and IgG4-RD has since been strongly disputed.^20^ Similarly, lactoferrin, pancreatic secretory trypsin inhibitors^21 22^ and pancreatic trypsinogens^22^ have been reported to be associated with AIP, though all of these have lacked specificity or sensitivity to IgG4-RD, and the nature of the autoantibody has not been further examined.

Notwithstanding these shortcomings, there is compelling evidence of extant autoantigens in disease. The passive transfer of purified human immunoglobulins (IgG1 and IgG4) from people with active IgG4-RD into neonatal mice led to binding, and subsequent damage to exocrine organs (salivary gland and pancreas).^23^ Using cloned immunoglobulins from IgG4-RD patients’ dominantly expanded plasmablasts in single cell sorted plasmablasts, investigators were able to demonstrate the secreted monoclonal antibodies to be self-reactive versus a cytosolic cellular component.^10^ However, the identity of the cytosolic antigen in this study was not determined.

Thus, the identification of specific antibodies against a cytosolic target by Hubers is consistent with findings from others.^10^ However, why Annexin A11 would be targeted still demands explanation. It is an intracellular protein, so it would follow that the antigen would only be presented to the antibody in the event of cellular damage. Furthermore, there is no obvious clue as to how binding to Annexin A11 would influence pathology. Annexins are a family of calcium-dependent phospholipid-binding proteins—their role in a fibroinflammatory disease is unclear, though, as the authors point out, autoantibodies against Annexin A11 have also been demonstrated in systemic lupus erythematosus, systemic sclerosis and primary antiphospholipid syndrome.^24^

Some caution must remain about the observations because there is a lack of validation in an external cohort, and of the 50 patients with IgG4-RD, only 9 had sera that reacted to Annexin A11—the authors have rightly not presented this as a diagnostic test. Validating this selective finding and understanding whether and how it relates to disease pathogenesis is key to appreciating the long-term impact of the work. The same group have published elegant work demonstrating dominant IgG4+ B-cell receptor clones accurately distinguish patients with IAC and AIP from primary sclerosing cholangitis and cholangiocarcinoma.^13^ IgG4+ B-cell receptor clones constituted a greater proportion of the total IgG+ repertoire in patients with IgG4-RD—and there were multiple clones, suggesting that there may be multiple antigens driving the observed response. Furthermore, the longitudinal examination of plasmablast clones in patients who have relapsing disease after successful initial treatment with B-cell depletion therapy (rituximab) has shown that the circulating plasmablasts that re-emerge are clonally distinct and exhibit enhanced somatic mutation compared with the initial circulating plasmablasts in the same patients.^10^ It is unclear whether the same antigens are recognised during the initial disease process and at the time of relapse. This raises the question as to how to measure and understand the significance of specific antigens in the disease process.

The causal relationship between the observed immunoglobulin response and pathology remains another hole in our knowledge. Hubers demonstrates that IgG4 from sera of patients diminishes IgG1 binding to Annexin A11.^11^ The authors speculate that IgG4 may act to dampen the IgG1-mediated pathogenesis in response to Annexin A11 binding—supporting an anti-inflammatory role for IgG4 in IgG4-RD. This follows published work in 2016, where the investigators demonstrated the pathogenicity of circulating IgG in patients with IgG4-RD by the passive transfer IgG1 and IgG4 into neonatal mice by subcutaneous injection.^23^ Both IgG1 and IgG4 bound to murine pancreas and salivary glands and led to subsequent damage, yet the effect was more pronounced in mice injected with patient IgG1. However, the potent pathogenic effects of patient IgG1 were significantly blunted by simultaneous injection of patient IgG4. It seems as though IgG4, though pathogenic, can competitively bind to target organs in preference to IgG1 and dampen its exaggerated effects.

The tolerogenic effects of IgG4 in IgG4-RD remain speculative, although they are well established in other disease settings.^25^ Peculiarities of the structure of IgG4 subclass lend itself to an anti-inflammatory role. Weaknesses between the heavy chains allow it to dissociate as two half molecules and associate with another IgG4 half molecule—a phenomenon known as ‘Fab arm exchange’.^8^ This results in a functionally monovalent IgG with bispecificity—thereby restricting the formation of immune complexes. Moreover, IgG4 has poor affinity to Fc-gamma receptors on effector cells, and to C1q—rendering them unable at activating the classical complement pathway.^9^ The classic example of IgG-mediated immune tolerance is seen in beekeepers, which are naturally exposed to high levels of bee venom allergen. Tolerant individuals secrete high concentrations of venom-specific IgG4 as opposed to other IgG subclasses and IgE.^26 27^ IgG4 is thought to competitively bind to the allergen in preference to IgE, thereby inhibiting IgE-mediated immune complex formation and mast-cell activation. Conversely, the immune-dampening effects of IgG4 can interfere with beneficial humoral responses. Melanoma cells secrete interleukin (IL)-4 and IL-10 to direct a modified T helper cell-2 response.^28^ Secreted IgG4 can block the effects of melanoma-specific IgG1, which are potent activators of macrophages and thus capable of initiating tumour cell death. Consequently, tumour-specific serum IgG4 concentrations correlate to mortality.^29^ The relevance of IgG4 in the pathogenesis of IgG4-RD remains confusing. Though serum levels do not faithfully correlate to disease activity, the excess of circulating IgG4 in active disease intuitively argues against a protective role. We cannot yet extrapolate whether IgG4 antibodies are primarily pathogenic, protective or neither.

Seemingly, IgG4-RD is, in part, antigen driven, and Hubers’ article in this issue of *Gut* lends a significant boost to the evidence base. How this reflects host risk continues to evolve and of note are conference reports now exploring host genetic risk, in robustly collected populations. A genome-wide association study of IgG4-RD, for example, has been performed in a Japanese population (Terao *et al*. International Symposium of IgG4-RD and Fibrosis, Feb 2017), and this reported three susceptibility loci consistent with antigen-driven disease: *HLA-DRB1*, *HLA-A* and *FCGR2B*, the latter encoding a low affinity receptor for IgG.

IgG4-RD is as much related to IgG4, as it is to clonally expanded B-cell populations, and an array of T cell subsets, although it is not classically preneoplastic, with plasmablast expansion being oligoclonal, not polyclonal. New technologies have increased our understanding of the changes in B-cell populations in different stages of the disease, but focus is now shifting additionally to delineating the role of T cells, in particular T follicular helper (Tfh) cells. Tfh cells help B cells and augment germinal centre development. They play a critical role in immunoglobulin somatic hypermutation and class switching of antibodies.^30^ In IgG4-RD, they are increased in numbers both in circulation and at sites of active disease, with increased expression of effector cytokines and regulators.^31^ In particular, the Tfh2 subset is associated with disease activity, the number of affected organs, B-cell differentiation and serum IgG4 levels, and responds to glucocorticoid treatment to parallel clinical improvements.^32 33^ As with the previously mentioned clonally expanded cytolytic CD4+ T cells, Tfh cell and B cell interactions are critical to the disease process. Type 2 Tfh cells seemingly activate B cells, which become memory B cells or plasmablasts. Activated B cells and plasmablasts can present antigen to CD4+ cytotoxic T cells at sites of disease.^34 35^ Supporting this, of course, is the apparently very positive impact of rituximab (anti-CD20) as a therapy.^36^

**Figure 1:**
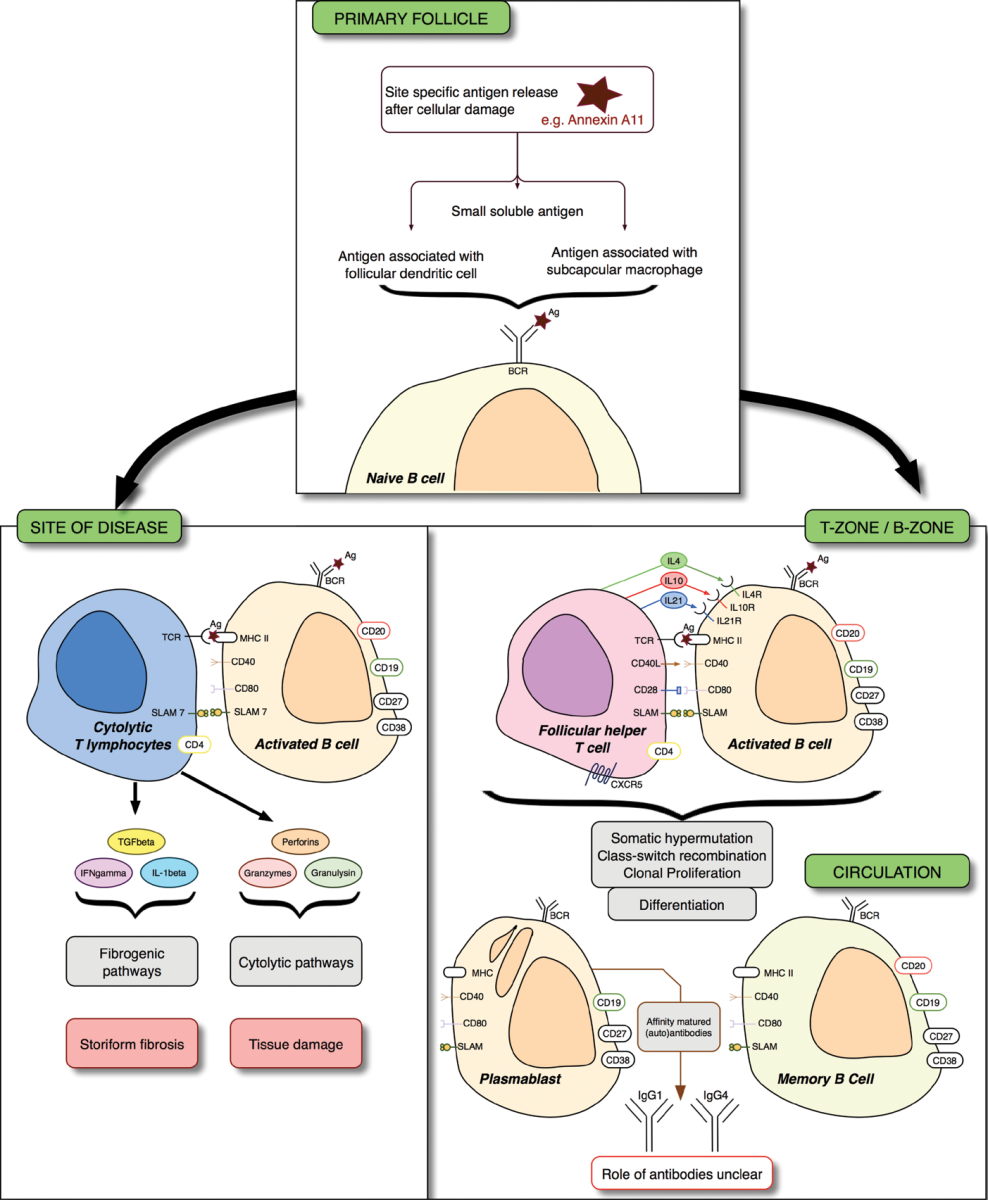
Immunoglobulin G4-related disease (IgG4-RD) and immune pathways to therapy. Naïve B cells are activated by exposure to antigens. In tertiary lymph nodes or in tertiary lymphoid tissue within an affected tissue, T follicular helper (Tfh) cells help B cells differentiate into antibody secreting cells. Interleukins (IL) 4, 10 and 21 are critical to B-cell affinity maturation, class switching and clonal expansion. At the site of disease, B cells are thought to interact with cytolytic T cells by the mutual expression of signalling lymphocytic activation molecule 7 (SLAM7). These effector T cells secrete profibrogenic cytokines that may be critical to subsequent storiform fibrosis, and cytolytic enzymes. The exact nature of the B cell to cytotoxic T cell interaction is still unclear. Therapy targeting CD20 (rituximab) leads to a reduction of plasmablasts as a consequence of killing their parent cells; plasmablasts do not express CD20. XmAb587 1 is a monoclonal antibody therapy that targets CD19 and enhances FcγRIIb-mediated inhibition—a receptor that inhibits B-cell function. A phase II trial examining the effect of XmAb5871 in IgG4-RD has completed enrolment. Elotuzumab leads to SLAM7-induced antibody directed cellular cytotoxicity in multiple myeloma. The utility of elotuzumab in IgG4-RD is currently only theoretical. Other therapies that may interfere with the pathogenic process are beyond the scope of this article, but could include therapy targeting the BAFF APRIL pathway (belimumab, atacicept); BAFF is critical for B-cell survival. Ag, antigen; APRIL, a proliferation-inducing ligand; BAFF, B-cell activating factor; BCR, B-cell receptor; CXCR5, chemokine receptor type 5; MHC, major histocompatibility complex; TCR, T cell receptor.

IgG4-RD, while very rare, remains an informative disease to study. Therapeutically, it portrays an immune-mediated disease with treatment options beyond corticosteroids, thanks to a greater understanding of the underlying pathophysiology (figure 1). Scientifically, it describes an evolving immunobiologic process, the unravelling of which will aid the understanding of all autoimmune disease.
